# Assessing farmers’ knowledge of environmental policy along the Ayeyarwady River: Strides towards the Indian Ocean marine life safety

**DOI:** 10.1016/j.heliyon.2024.e35503

**Published:** 2024-08-06

**Authors:** Lazarus Obed Livingstone Banda, Chigonjetso Victoria Banda, Jane Thokozani Banda, Thin Thin Hlaing, Eretia Mwaene

**Affiliations:** a Nalikule College of Education, Faculty of Science, Department of Computer Studies, Lilongwe, Malawi; b University of Malawi, Political & Administrative Studies, Zomba, Malawi; c Ministry of Education, Higher Education, Malawi; d Huazhong University of Science and Technology, College of Public Administration, China; e Beijing Institute of Technology, Graduate Education, Beijing, China

**Keywords:** Ayeyarwady river, Environmental policy, Marine life conservation, Sustainable agriculture, Policy implementation, Farmer awareness, Water pollution

## Abstract

The Ayeyarwady River Basin in Myanmar is grappling with severe environmental challenges, including soil erosion and water pollution, primarily driven by unsustainable agricultural practices. This study aims to evaluate farmers' awareness of environmental policies and identify barriers to their effective implementation. In-depth interviews were conducted with 45 stakeholders, encompassing farmers, government officials, and researchers. The findings highlight a significant lack of policy awareness among farmers, exacerbated by socio-cultural, economic, and institutional obstacles. These barriers impede the successful application of environmental policies, perpetuating environmental degradation. The study advocates for integrative strategies that encompass education, community engagement, and adaptive policy frameworks to address these complex issues. Detailed policy implications are provided, offering insights into potential solutions for enhancing the region's environmental governance and sustainable development. This research contributes to understanding the critical interplay between policy awareness and ecological management, underscoring the importance of targeted interventions to mitigate environmental threats.

## 1 Introduction

The Ayeyarwady Basin faces significant environmental challenges due to unsustainable agricultural practices that lead to soil erosion, pollution, and water quality degradation. Myanmar's ecological regulations are designed to address these issues, yet various socio-economic and cultural barriers hinder their effectiveness (see Table 1).

**Table 1:** Sample characteristics and coding.

Category	Details
Sample Composition	23 males, 22 females
Participant Roles	9 large-scale farmers, 24 small-scale farmers
Government and Research Participants	4 from the Ministry of Environment, 3 from the Ministry of Agriculture, 3 researchers, 2 from the Ministry of Information
Age Distribution	Most aged 30–37, fewest aged 19-21
Data Coding	271 initial codes, collapsed to 16 memos and top-applied codes
Top Co-occurring Codes	7 among government officials
Primary Codes	16 from government representatives and scholars
Central Themes	1. Awareness of policy presence 2. Comprehending the policy details 3. Comprehending the link between policies, agriculture, and ecology

Despite these regulations, there is a notable gap in understanding how effectively these policies are communicated and implemented at the grassroots level. Previous studies have highlighted the general environmental challenges in the region and the importance of policy frameworks. However, there is a lack of detailed research on farmers' awareness and understanding of these policies and the specific barriers that prevent successful implementation.

This study aims to assess farmers' awareness of environmental policies intended to mitigate these issues and to explore the barriers to effective policy implementation. By focusing on the Ayeyarwady River Basin, the research seeks to identify the specific gaps in policy awareness and the factors that impede successful implementation. The study also examines the broader implications of these findings for environmental policy and sustainable development.

Key terms such as "farmers' awareness" and "farmers' understanding" are central to this research. Farmers' awareness refers to the extent to which farmers are informed about existing environmental policies and regulations. Farmers' understanding goes a step further, encompassing not only awareness but also the depth of knowledge and comprehension of how these policies impact their agricultural practices and the environment. Defining these terms in relation to the study's objectives helps to avoid ambiguity and provides a clear framework for analyzing the data.

The significance of this study lies in its potential to inform policy development and enhance environmental sustainability in the Ayeyarwady River Basin. By identifying the barriers to effective policy implementation and proposing integrative strategies to address these challenges, this research contributes to the broader discourse on sustainable development and ecological conservation. Addressing the identified research problem can lead to improved policy frameworks that are both environmentally sound and culturally attuned.

In conclusion, this study addresses a critical gap in the understanding of environmental policy implementation in Myanmar. By examining the specific challenges faced in the Ayeyarwady River Basin, the research offers valuable insights into the interplay of socio-cultural, economic, and institutional factors that influence policy efficacy. These insights can inform future policy development, contributing to the sustainable management of natural resources and the achievement of broader environmental and developmental goals [[1], [2], [3]].

## 2 Challenges of policy implementation

The persistent deterioration in environmental conditions remains a global concern, with no nation having attained sustainable environmental practices. Since 1970, the world's ecological footprint, indicative of resource utilization, has surpassed the planet's sustainable capacity, while biodiversity indices have plummeted by over fifty percent [4,5]. Concurrently, greenhouse gas emissions are escalating, exacerbating the repercussions of global warming, with attendant challenges in both impacts and the fairness of mitigation policies [6]. Air pollution remains a critical issue, claiming millions of lives worldwide, with recent exacerbations observed particularly in some areas of South-East and East Asia. Notable deficiencies in environmental policy implementation are pronounced, particularly within developing economies [[7], [8], [9]]. Consequently, a burgeoning discourse has advocated for a novel economic paradigm centered on 'degrowth' and social-ecological transformation. These developments underscore the inadequacy of prevailing sustainability initiatives in halting the overarching decline in environmental quality [10].

Policy formulation should not be considered an isolated endeavor [11]. It demands bending to diverse operational environments [11,12]. Due to specific economic conditions and legal frameworks, each country faces unique challenges in implementing policies. Studies highlight numerous instances where ecological policies have failed across various global contexts. In Australia, sustainability initiatives like the Melbourne 2030 and Sydney's 2005 Metropolitan Strategy have struggled to meet environmental goals [10]. In Botswana, many policies, including the Tribal Land Act on Agricultural Development, have failed to realize sustainable outcomes in managing agriculture and land [13]. Similarly, Canada's Endangered Species Protection Act is noted for its failure, primarily due to economic interests outweighing environmental conservation efforts [10,14]. China's environmental policies, such as the Green GDP initiative and the National Environmental Model City Programme, have faced significant implementation challenges [10,15]. In the United States, foundational environmental legislations like the Clean Air Act and Clean Water Act have encountered various obstacles that hinder their effectiveness [10,16]. These examples reflect a common theme of structural and implementation challenges, including economic conflicts, inadequate governmental capacity, and poor communication strategies, which collectively impede the success of ecological policies [10]. The challenges leading to these failures include institutional, financial, behavioral, technical, and cultural barriers and policy coordination and monitoring issues. Understanding these barriers is crucial for effective policy implementation across different governance levels and regions [17,18].

Institutional challenges involve inefficient government or organizational structures [11,12] stemming from bureaucratic inefficiencies, lack of coordination among departments, and insufficient political will. Institutions play a crucial role in addressing climate change through the framing, implementing, and monitoring of policies, with political commitment being a critical determinant of success [19]. For instance, in the Ayeyarwady region, bureaucratic inefficiencies and limited political engagement hinder the lead to unchecked industrial pollution.

Financial barriers include insufficient funding, slow infrastructure development, and diminishing the effectiveness of policy implementation [10,12]. Economic capacity is essential for the successful design and execution of policies. For example, in Myanmar's Delta region, inadequate funding has delayed the construction of crucial flood defense systems, thereby increasing vulnerability to floods and obstructing sustainable development efforts [18].

Resistance to change is another threat: Farmers cling to traditional methods due to distrust and unfamiliarity with innovative practices despite the benefits of integrated pest management and bio-fertilizers. Such resistance is common in Sagaing and Kachin states. This psychological barrier hampers sustainable agricultural development by impeding the adoption of eco-friendly practices and can obstruct policy implementation [17]. This resistance can be due to the collapse of financial schemes or incentives that support projects [19].

Besides, in rural Myanmar, advanced water purification technologies have seen low adoption rates because local communities are unfamiliar with their operation or maintenance, demonstrating the technical obstacles to policy implementation [1].

Energy policies can fail when there is poor coordination between different government bodies, or the organizational structure is not conducive to effective policy implementation. This might occur when multiple agencies with overlapping responsibilities fail to work harmoniously, leading to inefficiencies and unmet objectives [10,12,19]. Effective policy implementation necessitates robust coordination and monitoring, often compromised by a “top-down approach,” [20] a system where higher authorities make decisions without sufficient engagement or consideration of the unique challenges faced at local levels. Such a disconnection can result in poorly adapted policies to the specific needs of rural agricultural communities and debilitating implementations. For example, in Vietnam, this has caused significant gaps in policy application [17].

Additionally, strengthening “knowledge brokerage,” the process of managing the exchange of information among decision-makers to inform policy decisions, is crucial for bridging the information gap between policymakers, researchers, and practitioners [20]. It involves establishing continuous and constructive feedback channels incorporating local insights and empirical data into national policymaking. This process helps ensure policies are well-informed and practically applicable [20]. In Vietnam, the absence of such mechanisms has restricted the adaptation of agricultural practices to mitigate climate change impacts effectively. Promoting better integration of local knowledge systems and feedback loops [12] can significantly enhance the relevance and efficacy of policies [17].

Besides, a significant contributor to policy failure is procedural inconsistency, where policies change frequently with shifts in political leadership, creating a landscape of uncertainty that undermines the stability needed for successful energy projects [19].

Cultural and personal barriers critically influence policy implementation [20,21]. In Vietnam's Northern Highlands, for instance, the traditional practice of slash-and-burn agriculture is deeply intertwined with the cultural identity of ethnic minority communities. This practice, while culturally significant, leads to substantial environmental degradation. Policy interventions have been more successful when they incorporate local cultural understanding and engage community leaders, showing respect for traditional methods while introducing sustainable alternatives [10,20]. This approach has begun to alter perceptions, demonstrating the potential for culturally sensitive policy frameworks to facilitate the adoption of environmental policies [17].

On a personal level, resistance to change among farmers, especially the older generation in the Mekong Delta, poses a significant barrier to adopting innovative farming techniques such as bioengineering and crop rotation. Pilot projects demonstrating the tangible benefits of these practices, like improved yields and soil health, have helped reduce skepticism. These initiatives highlight the necessity of integrating personal experiences and demonstrated benefits into policy implementation strategies, ensuring that innovations are technically sound, socially acceptable [20], and practically beneficial [17].

However, despite some grounds on which policy success is measured, we need to realize that judging the success or failure of a policy is marred with subjectivity and depends on the angle from which the judgment is passed [12].

## 3 The O-ring theoretical framework

This study employs Michael Kremer's O-ring Theory as a foundational framework, offering insight into the fragility of complex systems where success hinges on flawless execution across all components. Inspired by the Challenger space shuttle disaster, the theory underscores the critical importance of meticulous execution in high-stakes environments. It posits that even minor errors in any system component can lead to systemic failures, emphasizing the interconnectedness of system components and the potential for minor errors to have outsized consequences. The core concepts of the O-ring theory include “low-level equilibrium pitfalls."

This refers to situations where a system or economy remains in a state of suboptimal performance due to inefficiencies or failures in its individual components. This concept emphasizes that minor errors within specific parts of a system can prevent it from achieving higher levels of productivity and overall effectiveness. It highlights the critical importance of meticulous execution across all components, as minor discrepancies can lead to substantial systemic failures.

From an academic perspective, the concept underscores the interconnectedness of system components and the potential for minor inefficiencies to significantly impact overall performance. Addressing these inefficiencies at all levels is crucial to avoid being trapped in a state of low-level equilibrium, where the overall system performance is compromised. This perspective stresses the need for comprehensive solutions that enhance the functionality of all parts of the system to achieve optimal performance.

In the context of policy and development, “low-level equilibrium pitfalls" describe scenarios where developing economies struggle to achieve substantial growth due to pervasive inefficiencies in governance, infrastructure, or institutional frameworks. This concept helps explain why some countries remain in a state of underdevelopment despite efforts to implement economic reforms. By recognizing the importance of addressing inefficiencies at all levels, policymakers can develop strategies that avoid these pitfalls and promote sustainable economic development. Our choice of this theory is inspired by our belief that each phase in the policy process is as vital for implementation.

However, the theory is not without criticisms. It may overly emphasize the necessity for perfection and overlook the resilience of larger systems where multiple redundancies can absorb the impact of small failures. In the practical context of this study, while the theory underscores the critical need for meticulous policy implementation, it is also essential to recognize that the human components of the system policy implementers and stakeholders can sometimes adapt or compensate for minor discrepancies.

Thus, applying the O-ring theory to the environmental policies along the Ayeyarwady River highlights both the necessity for comprehensive accuracy in policy execution and the need to build systems that are robust enough to handle imperfections without leading to systemic failures. This dual focus is essential for crafting policies that are both stringent in their expectations and flexible enough to adapt to on-ground realities.

### 3.1 Practical examples

To better understand the implications of the O-Ring Theory in real-world contexts, it is useful to examine practical examples where the interconnectedness of system components highlights the importance of comprehensive interventions. One such example can be found in the healthcare system of a developing country.

#### 3.1.1 Case study 1: healthcare system in a developing country

Consider a developing country's healthcare system, plagued by low-level equilibrium pitfalls. The system's performance is hampered by inadequate training of healthcare workers, insufficient medical supplies, poor infrastructure, and weak administrative processes. These individual inefficiencies collectively prevent the healthcare system from achieving optimal performance. For instance, even if a hospital receives state-of-the-art medical equipment, the lack of trained personnel to operate it effectively renders the investment futile. Addressing only one aspect of the problem without considering the interconnected nature of the system leads to persistent underperformance.

#### 3.1.2 Case study 2: education sector in rural areas

In rural areas, the education sector often faces low-level equilibrium pitfalls. Factors such as poorly trained teachers, lack of educational materials, inadequate school facilities, and limited access to technology contribute to a suboptimal educational environment. These inefficiencies create a cycle where students do not receive a quality education, limiting their future opportunities and perpetuating the region's socio-economic challenges. Comprehensive interventions addressing teacher training, infrastructure improvements, and resource availability are necessary to break this cycle and improve educational outcomes.

#### 3.1.3 Policy implementation in agricultural development

In agricultural development, low-level equilibrium pitfalls can occur when policies to improve agricultural productivity are not effectively implemented due to fragmented efforts. For example, a policy promoting advanced irrigation techniques may fail if farmers lack access to necessary equipment, training, or financial resources. Additionally, if supporting infrastructure, such as roads and markets, are not developed, farmers cannot benefit fully from increased productivity. A coordinated approach that addresses all these factors is essential to overcome the pitfalls and achieve sustainable agricultural development.

These examples illustrate how low-level equilibrium pitfalls manifest in different sectors and highlight the importance of addressing systemic inefficiencies comprehensively. By understanding and applying the concept, policymakers can develop more effective strategies to promote sustainable development and economic growth.

#### 3.1.4 Application of the O-ring theory to policy implementation challenges

The O-Ring Theory, which highlights the importance of the performance of individual components within a system, is highly relevant to the challenges faced in the policy domain under investigation in this study. This theory provides a useful framework for understanding and addressing the issues in policy implementation in Myanmar's agricultural and environmental sectors.

The interconnectedness of components is a core aspect of the O-Ring Theory, and it directly relates to the multiple interconnected elements involved in policy implementation. These components include government officials, farmers, infrastructure, and information dissemination systems. The theory underscores that the failure of even a single component can lead to overall system inefficiency. For instance, if government officials are not adequately trained or farmers lack awareness and understanding of the policies, the implementation process can falter, mirroring the systemic inefficiencies identified in the study.

Quality and execution are also crucial under the O-Ring Theory, which posits that high-quality execution is necessary across all components to ensure system success. Effective policy implementation in Myanmar requires high-quality execution in policy formulation, stakeholder engagement, communication, and enforcement. Any deficiency in these areas can compromise the overall effectiveness of the policies, as seen in the study's findings on the lack of comprehensive stakeholder involvement and communication challenges.

Resource allocation, another key tenet of the O-Ring Theory, suggests that resources should be allocated efficiently to ensure all components function optimally. In the context of this study, this means investing in education and training for both officials and farmers, improving infrastructure for communication and transportation, and ensuring adequate resources are available for policy enforcement and monitoring. The study highlights the need for more robust advocacy and awareness campaigns, which align with the theory's emphasis on resource efficiency.

Systemic inefficiencies and the concept of “low-level equilibrium pitfalls" from the O-Ring Theory are evident in the challenges faced by Myanmar's policy domain. The study highlights issues such as lack of awareness, poor communication infrastructure, and inadequate advocacy efforts, all contributing to systemic inefficiencies. These inefficiencies prevent the successful implementation of policies aimed at sustainable agricultural practices and environmental conservation.

The O-Ring Theory advocates for a comprehensive approach to system improvement, addressing all potential points of failure. This aligns with the study's findings that a holistic strategy involving stakeholder engagement, improved communication, and adequate resource allocation is essential for effective policy implementation. By recognizing the interconnectedness of components and addressing inefficiencies comprehensively, policymakers can develop more effective strategies to promote sustainable development and economic growth.

## 4 Design and methodology

The phenomenological approach in this study is rooted in the philosophical tradition established by Edmund Husserl, which emphasizes exploring how individuals experience and make sense of their world. The phenomenological approach in this study is a research design deeply rooted in the philosophical traditions established by Edmund Husserl and further expanded by Martin Heidegger and Maurice Merleau-Ponty. This approach is dedicated to understanding and describing the essence of lived experiences from the perspective of those who undergo them. At its core, phenomenology seeks to capture the subjective interpretations and meanings individuals attach to their encounters with the world, emphasizing the unique and personal nature of these experiences (Groenewald, 2018; Padilla-Díaz, 2015).

The central focus of phenomenological research is on the lived experiences of individuals, aiming to delve into the intricate ways in which people perceive and make sense of their experiences. Their beliefs and values shape each person's perspective and prior encounters, forming a distinctive lens through which they interpret new phenomena. By exploring these subjective interpretations, phenomenologists strive to uncover the richness and depth of personal narratives, thereby providing a comprehensive understanding of the phenomenon under study [22,23].

In phenomenological research, data gathering typically involves unstructured or semi-structured in-depth interviews, participant essays, focus group discussions, and field notes. The interview method allowed for the collection of nuanced, detailed data encapsulating the complexities of the farmers' and officers’ lived experiences. To ensure that the findings are authentically grounded in the participants' perspectives, we engaged in a process known as bracketing, where they set aside our own biases and preconceptions. This practice was crucial for maintaining the integrity of the participants' viewpoints and preventing the imposition of the researchers' assumptions onto the data [22,24].

Data analysis in phenomenological research involves the process of data explicitation, wherein researchers identify and extract themes and patterns from the collected data. This iterative process describes the phenomenon's essence by synthesizing individual experiences into a coherent and comprehensive understanding. Through this synthesis, researchers aim to develop a shared understanding that reflects the multifaceted nature of the phenomenon, capturing its complexity and depth from multiple perspectives [22,24].

Ultimately, phenomenological research design was particularly suited for exploring the meanings and interpretations that participants in the region of study attach to their experiences. By compiling and analyzing these subjective accounts, this study offers detailed and nuanced insights, providing a richer and more comprehensive understanding of the phenomenon being studied. This methodological approach highlights the individuality of each participant's experience and seeks to uncover the commonalities that contribute to a deeper, collective understanding of the phenomenon [22,24].

### 4.1 Sampling and sample size

Data was gathered through face-to-face semi-structured interviews with 45 participants, including 9 government officers, 9 large-scale farmers, 3 researchers, and 24 small-scale agrarians. This approach aimed to assess their awareness and understanding of the existing policies comprehensively. Participants were chosen using purposeful sampling methods [25]. Naypyidaw Union Territory was selected for its significance as the center of policy development and the base of operations for government personnel. The Irrawaddy region was chosen as the focal point for farming activities, mainly due to its higher number of small-scale farmers than large-scale ones [26,27].

A stratified sampling method was employed to enhance the sample size, ensuring minimal variance within each group and maximum variance between groups to enhance estimate accuracy [28,29]. The sampling frame for this study included farmers and public officers engaged in environmental and agricultural policy implementation in Myanmar. For large-scale farmers (mega farmers), a comprehensive database was obtained from the regional agrarian offices, which included all registered large-scale farmers. Participants from this list were randomly selected using a computer-generated random number list, ensuring everyone had an equal chance of inclusion.

Acquiring a similar database for small-scale farmers presented significant challenges. Unlike large-scale farmers, small-scale farmers often lack centralized registration and systematic record-keeping. We relied on local agricultural offices, community leaders, and farmer associations to construct a representative sample of small-scale farmers. This approach allowed us to identify and recruit a diverse and representative sample from the Ayeyarwady River Basin despite the ad hoc nature of the process.

Initially, the sample size was 30, comprising 6 public officers, 4 large-scale farmers, and 20 small-scale farmers. This number balanced data saturation with practical considerations like time and resource constraints. All farmers were from the Ayeyarwady River Basin, ensuring a focused study on this specific rural region.

Public officers were also drawn from a comprehensive database provided by their respective ministries, including the Ministry of Information, the Ministry of Agriculture, and the Ministry responsible for natural resource and environmental protection. Each officer in the database was assigned a unique identification number, and a random number generator was used to select participants, ensuring equal chances of inclusion. We categorized stakeholders relevant to this study as officers from these ministries at the policy level, reflecting their critical roles in policy formulation and implementation.

After analyzing the initial data from these 30 participants, the need for additional insights became apparent, leading to an increase in the sample size to 45 participants. The extra 15 participants were carefully sampled to avoid including those from the initial cohort, preventing priming effects and ensuring data integrity. Rigorous cross-referencing of participant lists ensured that none of the initial 30 participants were included in the additional sample, providing fresh perspectives free from prior influence.

The inclusion criteria for farmers required active engagement in farming for at least the past five years, ensuring substantial experience and insight into the agricultural sector. Public officers included in the study had at least two years of experience in policy implementation, providing knowledgeable perspectives on policy-related issues.

Additional participants were pre-selected from the same strata as substitutes to address non-response and dropout rates. Follow-up calls and reminders were used to maintain high response rates, and any dropouts were promptly replaced to preserve the sample's representativeness.

While the methodology employed in this study provides valuable insights into the challenges of policy implementation in Myanmar, several potential limitations must be acknowledged. The initial sample size of 30 participants, later increased to 45, may still be considered relatively small for broad generalizations about the entire population of farmers and public officers in Myanmar. To address this limitation, a stratified random sampling method was used to ensure the sample was representative of different sub-groups within the population, capturing diverse perspectives and experiences.

Qualitative data collection methods, such as semi-structured interviews, are inherently subjective and may be influenced by researcher biases or participants' willingness to share openly. Furthermore, qualitative data can be challenging to generalize to larger populations. A rigorous and systematic data collection and analysis approach was followed to mitigate these constraints. Interviewers were trained to maintain neutrality and avoid leading questions. Semi-structured interviews allowed for in-depth exploration of participants' experiences while maintaining a consistent framework for comparison. Data triangulation, combining insights from interviews with policy documents and observational data, enhanced the validity and reliability of the findings.

Geographical diversity and logistical challenges in reaching remote areas of the Ayeyarwady River Basin may have limited the ability to include participants from the most isolated regions, potentially underrepresenting farmers' perspectives in these areas. To address these challenges, local agricultural offices, community leaders, and farmer associations were utilized to identify and recruit participants from various locations within the basin. Where direct access was impossible, alternative methods such as telephone interviews ensured voices from remote areas were included.

Non-response or dropout rates can impact the sample's representativeness and the data's completeness. To mitigate this risk, additional participants were pre-selected as substitutes, and follow-up calls and reminders encouraged participation. Any dropouts were promptly replaced with participants from the same strata to maintain representativeness.

Selection bias could occur if certain groups were more willing or available to participate than others, potentially skewing the results. This was addressed using a random selection process within each stratum and ensuring diverse representation. Efforts were made to engage participants from various socio-economic backgrounds and geographic regions to minimize the impact of selection bias.

The study aimed to ensure a robust, representative, and ethically sound methodology by acknowledging these limitations and implementing strategies to mitigate their impact. These efforts enhance the reliability and validity of the findings, providing a comprehensive and nuanced understanding of the challenges and opportunities in policy implementation in Myanmar. The methodology, while not without its limitations, offers a valuable framework for exploring complex policy issues and informs future research and practice in this area.

We used stratified sampling to sample the individual officers. We categorized stakeholders relevant to this study as officers at the policy level, coming from the Ministry of Information, Ministry of Agriculture, and Ministry responsible for natural resource and environmental protection. Since public officials carry out national policy formulation processes in teams, for example, members of the parliament, senators, or whichever structure it be, depending on the country's governance structures, we needed to get a minimal number of respondents from this group. Such organized groups conduct activities based on well-established and coordinated protocols and procedures. We subcategorized them based on the relevant ministries in line with the study objectives. Those from information were included for policy implementation, which entails effective information diffusion, which is the core ministry responsible for communication.

### 4.2 The rationale for choosing these categories of participants

The participants selected for this study—public officers, mega farmers, and minor-scale farmers—are particularly well-suited to achieve the research objectives, as they offer diverse perspectives essential for a comprehensive analysis of the ecological protection policy in Myanmar. Here's how each group contributes explicitly to the study objectives:

1. Civil Servants: By involving public officers from relevant ministries, the study gains crucial insights into the policy formulation, objectives, and expected outcomes. These are key to understanding how policies are communicated and implemented at the national level, and their perspective is vital for assessing whether the policy framework is designed to support sustainable agriculture effectively. This directly relates to the study's objective of analyzing farmers' understanding and implementation of ecological protection policies.
2. Large-Scale Farmers: This category of farmers typically have significant agricultural operations and may have more direct interactions with policymakers and more substantial resources and capacities to implement ecological practices. Their experiences and responses to policy can highlight the effectiveness of the policy's design and its adaptability to large-scale farming operations. This is critical for the study's objective to explore how different scales of farming operations implement ecological protection policies and perceive their benefits.
3. Small-Scale Farmers: Including subsistence or marginal farmers, who comprise most of the agricultural sector of the Irrawaddy region, is crucial for understanding the ground-level impact of ecological policies. These farmers' experiences are essential to evaluate the practical challenges and opportunities that arise from policy implementation. Their input is critical in addressing the objectives of appreciating the policy's impact on sustainable practices and the real-life circumstances affecting policy implementation among the wider farming community.

The stratified and purposeful sampling approach guarantees that the study encompasses a wide array of experiences and insights across different levels of farming operations and policy influence. This methodological choice is particularly adept at addressing the complex interplay between policy formulation at the national level and its implementation at the grassroots level. It provides a nuanced understanding of how policy is interpreted and enacted across different farming scales and how these actions align with national sustainable agricultural goals.

Overall, carefully selecting these participants is directly aligned with the study's goals, enabling a detailed investigation of the macro and micro impacts of ecological protection policies in Myanmar. It allows the research to effectively address how these policies influence sustainable agricultural economic development, farmer awareness and knowledge, perceived benefits, and the practical implementation of policy among the farming communities.

### 4.3 Data collection

The interview method was selected as the most suitable approach for this research. Three experts tested the interview questionnaire for face and content validity before pilot-testing it among five stakeholders for perfecting irregularities. Interview data was collected by trained research assistants versed in both the native Myanmar language and English. The questionnaire for the semi-structured interviews was in both languages to enable maximum understanding by the participants. Voice recorder applications on smartphones were simultaneously used with informed consent from the interviewees, and note-taking was scribbled on iPads to ensure no details were lost during transcription. After that, the data was transcribed into English by bilingual expert transcribers before we imported the same into Atlas 23 for analysis. These interviews were analyzed through thematic analysis using Atlas version 23. Coding was done manually to capitalize on the strengths of the human ability to understand gestural communication.

One of the primary purposes of interviews is to acquire first-hand information from participants. Interviews offer the chance to collect extensive and comprehensive data on a matter of interest straight from the informant [30]. This original data can be utilized to investigate various research queries and hypotheses.

Furthermore, we chose to use in-depth interviews since this approach additionally acts as an opportunity for the researcher and participant to exchange ideas. Conversation allows the researcher to obtain a more thorough comprehension of the participant's viewpoint and insights. The participant can benefit from the investigator's know-how and field-specific knowledge [29]. When conducting qualitative research, such interviews can clarify the complexity of participants' experiences and perspectives, resulting in a deeper understanding of the topic of study. We also preferred this approach because it can detect trends and patterns inside the data. Through the analysis of responses from many respondents, investigators can discriminate common themes and trends that enhance their research findings. These patterns can also highlight potential areas for further investigation and help formulate new hypotheses for future studies. Moreover, the semi-structured interview approach can be tailored to the specific requirements of the research project, enabling researchers to ask follow-up questions or investigate unexpected emerging topics, thereby achieving a more thorough understanding of the research subject [30].

However, we realize that interviews are not only a good approach to data collection but are also associated with some challenges. When adopting interviews for this study, we were aware of the following difficulties, which influenced us to mix the qualitative approach with quantitative data. Besides, interviews can be resource-intensive and time-consuming.

Additionally, the interviewer's communication can influence participants' readiness to share their experiences or perceptions. Conducting interviews can be time-intensive and require significant resources, especially when numerous interviews are involved. To address this challenge, we utilized a manageable sample size.

Furthermore, this method may be vulnerable to interrogator bias, where the interviewer's dogmata may affect the phrasing of questions or the interpretation of responses, potentially resulting in inaccurate or incomplete data. They may be subject to social desirability bias, in which participants provide socially acceptable responses instead of their true thoughts and emotions. Participants may be inclined to give socially acceptable reactions instead of their true thoughts and feelings to please the interlocutor or avoid social disapproval. This can result in inaccurate or insufficient data. This challenge was handled from the start by ensuring the interviewees' confidentiality and anonymity, in which no response was right or wrong. The communication style of the interviewer can influence participants' readiness to share their experiences or viewpoints. Therefore, we ensured that our interviewers were thoroughly trained to recognize and address these challenges. The interviewers were professional data collectors, equipped to maintain awareness of potential biases and to facilitate open, unbiased conversations.

### 4.4 The duration and procedure of the interview

The interview process was designed to ensure alignment with ethical standards and facilitate in-depth exploration of farmers’ understanding of the ecological protection policy within the Irrawaddy Basin region. Semi-structured and open-ended questions were employed, avoiding leading or yes/no inquiries except when necessary. This approach allowed participants to express their perspectives freely and provided researchers with rich qualitative data. Additionally, participants were informed at the outset that the interview would last between 45 min to 1 h, acknowledging and respecting their time commitments. However, flexibility was maintained to accommodate extended engagement periods, ensuring that participants had ample opportunity to share their insights comprehensively.

Ethical considerations were paramount throughout the study, with adherence to the Declaration of Helsinki and its ethical principles. Prior to each interview session, participants were provided with detailed explanations of the study's objectives, procedures, and potential implications, ensuring informed consent and respect for participants' autonomy. Overall, the interview process was conducted in a manner that prioritized ethical standards, respect for participants' time, and the generation of meaningful insights into farmers' understanding of ecological protection policy in the Irrawaddy Basin region.

## 5 Findings

Table one summarizes sample characteristics and coding.

### 5.1 First theme: awareness of the presence of the policies

A public servant noted that less than half of the farmers are aware of the public policy aimed at protecting natural resources in the country. The participant emphasized the necessity for increased advocacy and educational efforts to improve policy implementation.

“Less than half of the farmers may understand these policies. Most farmers are unaware, leading to a substantial misunderstanding about the government's intentions behind introducing these policies.” (A government officer).

A subsistence farmer mentioned being aware of public policies related to ecological conservation but did not comprehend why these regulations should have a significant impact on the agricultural sector. She expressed her limited knowledge of the policy in the following statement:

“I have heard about some rules, but I do not understand them. Basically, I am not familiar with the specifics." (A female subsistence farmer).

“There are numerous laws concerning environmental protection, but I am not familiar with the details. I know there are restrictions on how land can be used, but I lack knowledge about the specifics of these policies." (A male subsistence farmer).

In addition to their own lack of awareness, some farmers suggested that many others likely subscribed to the same situation. Echoing the perspective of a public officer, one farmer asserted that, despite the existence of these policies, only a small number of farmers might be aware of them. The following quotation elucidates his viewpoint:

It appears that very few individuals are aware of the existence of these policies. Nonetheless, there is a pressing need for greater education about the policy, as it would significantly enhance the protection of natural resources.

Despite his ignorance of the extant policies, he believes those aimed at safeguarding ecological artifacts and resources could be an excellent asset for sustainable agrarian activities contributing to economic development. He insists that public policy would significantly intervene in ensuring continuity in productive agriculture and sustainable development. The farmer had the following to say:

Currently, farmers' excessive fertilizer use is causing adverse effects on the soil and damaging natural resources. Increased knowledge and proper implementation of these policies would be beneficial. Unfortunately, many farmers do not have a clear understanding of these issues.

### 5.2 Second theme: comprehending the policy

The theme concerning farmers' awareness of public policies related to environmental protection revealed significant disparities in awareness levels and underscored the importance of extension services, traditional knowledge, and local initiatives.

The theme of farmers' awareness of public policies regarding environmental protection highlighted notable disparities in understanding. These differences are influenced by various factors, including location, education, and access to information. Addressing these awareness gaps is critical, as greater engagement with farmers is required. Due to insufficient awareness, many farmers perceive the policy as hindering their progress rather than recognizing its importance and benefits (official).

Several farmers supported the official's perspective, admitting that while they were aware of the policies' existence, they did not fully understand them. The following quotation reflects their sentiments:

Advocacy, lobbying, public support, awareness, misunderstanding, lack of knowledge, ignorance, access to information, balancing between environmental conservation, civic education, and farmers’ engagement in policy development were among the common issues that public officers concerning determinants of the role of agricultural economic development in promoting sustainable economic growth are significant.

Recognizing that each stakeholder perceives issues from their unique viewpoint, respondents stressed the importance of balancing agriculture and conservation, economic opportunities, climate resilience, resource scarcity, and socio-economic impacts. Some farmers have embraced environmentally beneficial practices, yet many fail to grasp their significance. These farmers often misunderstand the interconnectedness between agriculture, environmental health, and sustainable development.

### 5.3 Third theme: comprehending the significance of the ecological policy

Officers and farmers unanimously expressed concerns about the detrimental impact of abolishing environmental policy on agriculture's economic development. One female farmer noted,

“I do not understand the policy very well. We farm in permitted areas, but we have not been informed about why other areas are restricted. We comply because we are forced to avoid certain lands."

We do not understand the policy and are unaware of its details. No one informs us about it, and there is a lack of awareness. We need more information about the public policies aimed at safeguarding, protecting, and enhancing natural resources.".

“Many farmers harbor misconceptions about the policies, leading to considerable implementation challenges. This misunderstanding creates obstacles that make it difficult to achieve sustainable agricultural practices. If these policy implementation challenges are not addressed, the long-term viability of sustainable agriculture is compromised. As a result, the prospects for sustainable economic development are also jeopardized, putting both environmental and economic stability at significant risk." (officer).

“Therefore, as of now, I believe that farmers are still struggling to reconcile the requirements of environmental policies with the agricultural practices necessary for economic development in this region. This difficulty stems from a widespread lack of understanding and awareness about the policies and their implications." (officer).

### 5.4 Fourth theme: linking policy, agricultural productivity, and ecological conservation

Building on the previous theme, another respondent highlighted that the interconnections between agricultural practices and environmental policies are intricate and often challenging for the average farmer to comprehend fully. Some farmers perceive these government policies as obstructive to their agricultural economic activities. Despite these perceptions, the respondent emphasized fostering collaboration among farmers, policymakers, and the entire agro-food value chain. Such cooperation is essential for effectively addressing environmental challenges and ensuring that both agricultural productivity and environmental sustainability can be achieved.

Unsustainable agricultural practices by farmers can severely damage the environment. For example, when land experiences extensive soil degradation, substantial agricultural inputs are required for farmers to produce any yield, negatively affecting the regional economy. Furthermore, farming activities in vulnerable areas can lead to environmental degradation and increased flooding, which are detrimental to the overall economic stability of the region.

Building on previous quotes and themes, other farmers recognized the necessity of policies even though they do not fully understand them. The farmer expressed that achieving high yields is essential for economic progress. Still, the policies limit their ability to utilize certain lands, questioning how yield increases can be accomplished under such restrictions. They warned that removing these regulations would ultimately harm the nation's natural resources and economic development.

Additionally, the farmer highlighted the vital connection between environmental protection, agriculture, and economic stability. They noted that while many farmers focus primarily on agriculture due to its importance for their livelihood, it is crucial to strike a balance between agricultural practices, environmental sustainability, and economic objectives to ensure long-term success and ecological health.

A significant insight was shared by a public officer who expressed concerns about the lack of coordination in water safety monitoring and policy implementation. He noted that numerous organizations are tasked with ensuring water safety, yet they operate independently without coordination. This issue stems partly from resource limitations and partly from a lack of interest, especially where vested interests are involved.

When questioned about the potential benefits of abolishing the policies, there was a clear consensus that such regulations are crucial for agricultural productivity and should remain in place. One farmer articulated that repealing any environmental preservation policies would harm both agriculture and economic development. Another farmer suggested that, instead of abolishing these policies, the government should increase awareness campaigns to better inform the public about the procedures and regulations.

Regarding the relationship between environmental policy, agricultural production, and economic development in Myanmar, a senior public officer described it as complex and emphasized the necessity of sustainable agricultural practices and responsible resource management for long-term economic growth. They stressed the importance of raising awareness among farmers and providing adequate support for policy implementation. The officer highlighted that the interconnection between environmental policy, agriculture, and economic development is often misunderstood by many farmers, who perceive the policies as obstacles to their economic activities. However, enhanced collaboration among farmers, policymakers, and the agro-food value chain is essential to address enduring environmental challenges. While the officer understood this relationship, he acknowledged that many farmers did not.

Moreover, the respondent pointed out that negative perceptions of a country's environmental practices could deter foreign investment and tourism, thereby further impacting economic growth. Abolishing these policies would negatively affect sustainable agriculture. Thus, balancing agricultural practices with environmental protection is crucial to ensure a sustainable and prosperous future.

#### 5.4.1 Reasons for low levels of awareness and total ignorance in some cases about the policy, its importance, and its relationship with other factors of sustainable development in Myanmar

The preceding sections have meticulously examined the varying levels of awareness and understanding among officers and farmers regarding environmental policies. The findings highlighted significant gaps in knowledge and comprehension, with many stakeholders lacking a clear understanding of the policies' objectives and their implications for sustainable agricultural practices and economic development. These disparities underscore the complexity of implementing environmental policies in diverse socio-economic and cultural contexts.

Building on the themes of policy awareness, comprehension, and the interrelationship between agricultural practices and ecological conservation, it is crucial to delve deeper into the factors contributing to the observed low levels of awareness and, in some cases, complete ignorance of these policies. By identifying and analyzing these underlying reasons, we can better understand the barriers to effective policy implementation and develop strategies to enhance engagement and education among all stakeholders.

To begin with, officers identified several reasons for the low levels of awareness and, in some cases, total ignorance about environmental policies among farmers in Myanmar. They observed a higher level of awareness among officials compared to farmers, attributing this disparity to multiple factors. One policymaker advocated for a more integrated approach to policy implementation, emphasizing the necessity of involving all stakeholders from the earliest stages of policy formulation. He argued that grassroots participation in situational analysis, policy drafting, dissemination, and implementation is crucial for effective policy outcomes.

Further emphasizing this point, another respondent criticized the government's lack of commitment to advocacy and awareness campaigns. He highlighted the importance of ecological policies, stressing that agricultural activities could significantly harm the environment without them. This perspective underscores the positive relationship between environmental policies, agricultural production, and economic development, pointing to the government's inadequacy in educating the public about environmental protection.

A public officer from the Ministry of Information echoed these sentiments, noting that the geographical characteristics of Myanmar pose significant challenges to information dissemination. She explained that reaching remote farmers in the countryside is particularly difficult for advocacy and awareness purposes and for helping farmers transport their produce to better markets. This issue is compounded by the limited access to television and mobile technologies in some areas, which alienates farmers from essential information.

Some farmers reiterated these communication challenges, highlighting their difficulties in accessing information. One female farmer, citing her illiteracy, questioned the efficacy of disseminating policies through television in rural areas. She pointed out that mobile technologies in these regions are not as advanced as those in towns, further complicating access to information. She stressed the need for outreach efforts to be conducted at times convenient for the farmers; otherwise, they would remain ignorant of the policies.

Addressing the infrastructural challenges, a politician noted that the government's financial constraints impede the development of appropriate communication infrastructure. He mentioned that the Ministries of Communication and Transport and Public Works plan to improve rural roads and install a backbone for ICTs to facilitate better information dissemination.

Despite these low levels of awareness, public office respondents unanimously agreed that abolishing the environmental policy would negatively impact economic development in the agricultural sector. They emphasized the close link between environmental policies and economic growth, noting that these policies protect the natural resources essential for agriculture. Without such protections, irresponsible agricultural practices would harm these resources, ultimately undermining the long-term viability of the agricultural economy.

One official highlighted the government's lack of clear strategic objectives and robust success criteria. He pointed out that those responsible for raising awareness lack clarity on the processes and methods necessary for fulfilling their responsibilities effectively, further exacerbating the challenges in policy implementation.

The respondent identified various potential repercussions of eliminating environmental regulations on agricultural economic progress, such as ecological deterioration, diminished farming output, biodiversity decline, health issues, economic instability, and societal disruption. They underscored the importance of sustainable farming practices supported by these regulations, which are essential for ensuring food security, preserving natural resources, and fostering a stable economy. The necessity of balancing development and environmental stewardship was also highlighted. An official commented that:

The traditional approach to creating public policy involves listing possible scenarios, assigning probabilities to each, and calculating expected outcomes by considering their pros and cons. This technique works well when the options are well-defined and risks can be quantified. However, both business and public policy often function in intricate environments where predicting every potential situation is unfeasible, making it challenging to quantify probabilities, costs, and benefits accurately (officer).

The system's structure for public policy complicates or prevents the anticipation, assessment, and management necessary for effective policymaking, whether the aim is to improve public services or create new programs. Utilizing the traditional approach in such contexts often leads to failure or unforeseen outcomes. Moreover, increasing data, funding, or hiring more skilled experts usually does not resolve the issue. Enhancements to an already unsuccessful strategy have limited potential (officer).

One farmer expressed skepticism about abandoning familiar methods for uncertain ones, emphasizing that they would need incentives from advocates to consider making such a change.

## 6 Discussion

This scholarly examination synthesizes pivotal insights from the study “Assessing farmers' knowledge of environmental policy along the Ayeyarwady River," positioning its findings within the extensive discourse on policy implementation failures. It aims to elucidate how these findings correlate with and diverge from extant literature concerning policy implementation, environmental governance, and sustainable development, providing a rigorous analytical narrative.

### 6.1 Application of the O-ring theory in policy implementation

The application of the O-Ring Theory to the domain of policy implementation provides a profound understanding of the intricate failures that can undermine these processes. Analogous to the catastrophic failure of the Challenger space shuttle, precipitated by a minor fault in an O-ring, seemingly negligible oversights in policy execution can culminate in significant breakdowns in environmental governance. This theoretical framework underscores the imperative of meticulous execution across all dimensions of policy processes [31]. Failures in cultural comprehension, economic alignment, and political engagement can severely disrupt the successful implementation of policies.

The O-Ring Theory elucidates the necessity of addressing every component within a policy framework to ensure overall success. In the realm of environmental policy implementation, even minor lapses in stakeholder engagement, insufficient training for policy executors, or misaligned economic incentives can lead to substantial setbacks. The intricate interplay among these factors often escalates the complexity, thereby reinforcing the characterization of policy implementation as a “wicked problem" that defies simplistic solutions.

Addressing these multifaceted challenges mandates a holistic approach that acknowledges the interconnected nature of policy components. The theory posits that, similar to how the failure of a single O-ring resulted in the Challenger disaster, minor inefficiencies in policy processes can precipitate the failure of environmental governance initiatives. Consequently, meticulous attention to detail and comprehensive planning are indispensable in circumventing such pitfalls.

However, the pursuit of meticulousness in policy processes can introduce its own set of challenges. The increased bureaucracy and delays inherent in such an approach can hinder timely policy implementation, causing critical interventions to be postponed. Furthermore, the resource-intensive nature of detailed planning and execution can strain available resources, diverting them from other essential areas. The risk of over-complexity also looms large, as overly detailed policies can become difficult to understand and implement, deterring stakeholders from engaging fully and leading to poor compliance and execution. This complexity can create a rigid framework that lacks the flexibility to adapt to unforeseen circumstances or changing contexts, rendering the policies ineffective or irrelevant in dynamic environments.

Stakeholder fatigue is another potential consequence of an overly meticulous approach. Engaging stakeholders in a highly detailed process can lead to disengagement, especially if they perceive the process as burdensome without immediate benefits. This scenario can result in “paralysis by analysis," where decision-makers are so focused on perfecting every detail that they struggle to make timely decisions or take action. Additionally, meticulousness can sometimes lead to micro-management, where policymakers concentrate on minor details at the expense of strategic vision, stifling innovation and creativity.

Despite these potential drawbacks, the need for meticulousness in addressing the intricate failures in policy implementation remains critical. By focusing meticulous efforts on key areas with the most significant impact on policy outcomes and streamlining processes to reduce bureaucracy, policymakers can enhance efficiency and effectiveness. Prudent resource allocation ensures that critical areas receive sufficient investment without overstretching available resources. Moreover, designing policies with built-in flexibility to adapt to changing circumstances and incorporating feedback loops for continuous improvement can mitigate the risks associated with rigidity.

In conclusion, while the O-Ring Theory provides a robust framework for comprehending the complexity inherent in policy implementation within environmental governance, the pursuit of meticulousness must be balanced with practicality. By emphasizing outcome-based approaches and engaging stakeholders meaningfully without overburdening them, policymakers can ensure that meticulousness enhances rather than hinders the effectiveness of policy interventions. Recognizing and addressing these potential negative impacts allows for a nuanced approach that maximizes the overall efficacy of policy implementation.

### 6.2 Interplay of social, cultural, economic, and political variables

#### 6.2.1 Political variables

The intricate interplay of social, cultural, economic, and political variables significantly influences the effectiveness of policy implementation. Political dynamics play a quintessential role in shaping the trajectory and efficacy of policy implementation. These dynamics are often complicated by the influence of interest groups and bureaucratic inefficiencies, which can hinder the successful implementation of policies. The complexity of navigating political landscapes where policies face resistance or ineffective implementation due to conflicting interests and inadequate administrative capacities underscores the challenges inherent in the policy process [17,32].

Social and cultural factors also play a pivotal role in determining the acceptance and integration of policies within communities. Societal norms and behaviors are crucial in shaping social acceptance and cultural integration of environmental policies. Resistance to these policies often arises from their perceived disruption to traditional practices. Thus, policies must be designed to be environmentally sound, culturally attuned, and socially acceptable. The interaction between social norms and policy outcomes exemplifies the complex dynamics that characterize the policy implementation landscape, reinforcing the concept of policy implementation as a “wicked problem."

Economic factors are equally fundamental to the feasibility and sustainability of policy initiatives. Resource constraints necessitate prioritization that aligns with economic viability, often at the expense of long-term environmental and social benefits. The economic underpinnings of policy initiatives highlight the multifaceted nature of policy challenges, further reinforcing the notion of policy implementation as a “wicked problem." These economic constraints frequently lead to compromises that affect the efficacy of policies, emphasizing the need for economically viable and environmentally sustainable policies.

The challenges in policy implementation are starkly evident in the context of the Ayeyarwady River study. The endemic lack of understanding and engagement among local agrarian communities starkly impedes the practical application of environmental policies. This persistent challenge, particularly pronounced in developing economies, underscores the need for integrative approaches comprehensively addressing institutional, financial, behavioral, technical, and cultural barriers. Recognizing these challenges as elements of a “wicked problem" emphasizes the necessity for adaptive, multifaceted solutions that can navigate the complexities of policy implementation.

Disparities in environmental policy awareness among different stakeholder groups, notably between local farmers and academics or public officials, have significant repercussions for sustainable agricultural practices and economic development within the region. These gaps in awareness exacerbate environmental degradation, impede agrarian productivity, and undermine economic stability. Enhancing policy dissemination and educational initiatives to bridge the gap between national ecological objectives and localized agrarian practices is imperative. Ensuring that environmental policies are contextually relevant and practically implementable is crucial for their success.

This discourse reaffirms the critical necessity of understanding and addressing the intricate interplay of social, cultural, economic, and political variables to implement environmental policies successfully. Insights from the Ayeyarwady River study offer invaluable lessons for enhancing ecological governance and sustainable development strategies in comparable contexts. Highlighting the need for comprehensive, contextually informed policy frameworks that can adeptly navigate the nuanced challenges of policy implementation reinforces the perception of policy implementation as a “wicked problem." It demands innovative, integrative approaches for effective resolution.

### 6.3 Social and cultural factors

Societal norms and behaviors crucially influence social acceptance and cultural integration of policies. The resistance to environmental policies often stems from their perceived disruption to traditional practices, underscoring the imperative for policies that are not only environmentally sound but also culturally attuned and socially acceptable [12,17].

The successful implementation of policies depends significantly on how well they align with and respect existing social norms and cultural values. When policies are perceived as intrusive or misaligned with local traditions, they are likely to encounter substantial opposition, hindering their effectiveness.

The interaction between social norms and policy outcomes exemplifies the complex dynamics that characterize the policy implementation landscape as a “wicked problem." These dynamics underscore the multifaceted nature of policy acceptance, where the cultural context and societal behaviors must be considered in the design and execution of policies. For instance, in the context of environmental governance, policies that fail to incorporate local customs and practices are less likely to be embraced by the community. This cultural disconnect can lead to non-compliance, undermining the policy's intended outcomes.

Moreover, the need for cultural attunement extends to the ways in which policies are communicated and enforced. Effective policy implementation requires strategies that engage communities, respect their cultural contexts, and leverage local knowledge. This approach not only fosters greater acceptance but also ensures that policies are relevant and practically implementable. Engaging with community leaders and stakeholders in the policy development process can facilitate a deeper understanding of cultural nuances and promote more effective integration of policies.

Therefore, addressing the social and cultural factors in policy implementation involves a holistic approach that encompasses cultural sensitivity, community engagement, and respect for traditional practices. By aligning policies with societal norms and behaviors, policymakers can enhance the likelihood of successful implementation and achieve sustainable outcomes. This approach necessitates continuous dialogue with communities to adapt policies in ways that harmonize with cultural values while advancing environmental and developmental goals. The case of the Ayeyarwady River Basin study underscores the importance of such integrative strategies, where policies need to be culturally resonant to overcome resistance and foster sustainable environmental practices.

### 6.4 Economic factors

The economic underpinnings of policy initiatives are fundamental to their feasibility and sustainability. Resource constraints necessitate a prioritization that aligns with economic viability, often compromising long-term environmental and social benefits [10,12]. The intricate interplay between economic constraints and policy efficacy further highlights the multifaceted nature of policy challenges, reinforcing the notion of policy implementation as a wicked problem.

Economic constraints can also lead to trade-offs that affect the overall success of policy initiatives. For instance, limited funding may necessitate the prioritization of certain policy components over others, potentially leaving critical areas underfunded and underdeveloped. This can lead to gaps in policy implementation, where some aspects are effectively addressed while others are neglected, thereby reducing the overall efficacy of the policy.

Furthermore, the necessity for economic alignment often means that policies must be designed to attract investment and foster economic growth, which can sometimes be at odds with environmental and social goals. For example, policies aimed at industrial growth may lead to increased pollution and resource depletion if not carefully managed. This highlights the need for integrated approaches that consider economic, environmental, and social dimensions concurrently, ensuring that policies promote sustainable development without compromising any one area.

The concept of policy implementation as a “wicked problem" is reinforced by these economic challenges. Wicked problems are characterized by their complexity and the interdependence of various factors, making them resistant to straightforward solutions. In the context of economic factors, this means that addressing policy implementation requires a nuanced understanding of economic dynamics and the development of strategies that can navigate these complexities effectively.

### 6.5 Challenges in policy implementation

Structural and implementation challenges prevalent in the discourse on policy execution are palpably evident in the Ayeyarwady River study. The endemic lack of understanding and engagement among the local agrarian communities starkly impedes the efficacious application of environmental policies. This persistent challenge, especially pronounced in developing economies, underscores the exigency for integrative approaches that comprehensively address institutional, financial, behavioral, technical, and cultural barriers to achieve policy efficacy [[8], [9], [10]].

Institutional barriers often arise from fragmented governance structures and lack of coordination among agencies responsible for policy implementation, leading to inconsistencies and enforcement gaps. Financial constraints limit the scope and scale of policy initiatives, particularly in resource-scarce developing economies. Behavioral barriers, rooted in local attitudes and practices, necessitate targeted educational programs to shift perceptions and foster greater acceptance of environmental policies. Technical barriers, including inadequate infrastructure and training, restrict the capacity of communities to comply with and benefit from these policies. Cultural barriers further complicate implementation, as policies misaligned with local customs and traditions face significant resistance.

Wicked problems, characterized by complexity and interdependencies, resist simple resolutions. Addressing them requires a holistic approach that integrates various strategies and perspectives. Adaptive management, stakeholder engagement, continuous learning, and flexible policy frameworks are essential components of such an approach. These strategies can help navigate the intricacies of policy implementation, ensuring that policies are both well-designed and effectively executed.

### 6.6 Impact of environmental policy awareness on sustainable agriculture and economic development

Disparities in environmental policy awareness among different stakeholder groups, notably between local farmers and academics or public officials, have significant repercussions for sustainable agricultural practices and economic development within the region. These gaps in awareness exacerbate environmental degradation, impeding agricultural productivity and undermining economic stability. Thus, enhancing policy dissemination and educational initiatives to bridge the gap between national ecological objectives and localized agrarian practices is imperative, ensuring that environmental policies are contextually relevant and practically implementable [12].

This discourse reaffirms the critical necessity of understanding and addressing the intricate interplay of social, cultural, economic, and political variables to implement environmental policies successfully. Insights from the Ayeyarwady River study offer invaluable lessons for enhancing ecological governance and sustainable development strategies in comparable contexts, highlighting the need for comprehensive, contextually informed policy frameworks that can adeptly navigate the nuanced challenges of policy implementation. Acknowledging the complexity and interdependencies within these frameworks reinforces the perception of policy implementation as a wicked problem, demanding innovative, integrative approaches for effective resolution.

## 7 Conclusion

The study reveals significant awareness and understanding gaps. Most farmers are unfamiliar with these policies, which impact their agricultural practices and contribute to the river's pollution, affecting marine life in the Indian Ocean. Despite the policies' intent to foster sustainable agriculture and protect ecosystems, the lack of clear communication, education, and community engagement hinders effective implementation. The research highlights the need for enhanced policy dissemination and integration of local knowledge to bridge the gap between national objectives and local realities. This can lead to improved environmental governance and sustainable agricultural practices essential for the region's ecological and economic health. These findings underscore the complex interplay between environmental governance and sustainable development in Myanmar, aligning with literature that identifies knowledge gaps and implementation challenges as significant barriers to effective ecological policy [33].

The limited awareness among farmers about environmental policies is particularly concerning, given the pivotal role of the Ayeyarwady River in Myanmar's agricultural productivity and overall ecological health. This aligns with existing studies showing that effective environmental governance is crucial for conserving biodiversity and supporting sustainable economic growth [17,18]. However, our findings diverge by demonstrating a deeper level of disconnect at the grassroots, emphasizing that the lack of awareness is not merely institutional but is profoundly rooted in the socio-economic fabric of the rural communities.

Furthermore, this study contributes novel insights into how infrastructural deficiencies impact policy dissemination and enforcement, exacerbating the “policy-implementation gap” and framing environmental governance as a “wicked problem” that resists simple solutions and heavily relies on contextual nuances for resolution [33,34]. These insights underscore the need for strategies adapted to the specific realities of rural communities while addressing broader policy objectives, thereby enriching the discourse on sustainable development.

Policy implementation is frequently characterized as a “wicked problem," which describes issues inherently resistant to straightforward solutions due to their complex and interconnected nature [[35], [36], [37]]. This complexity arises from several factors, including the interdependencies between various social, economic, and environmental elements that can result in unpredictable outcomes when interventions are made. Additionally, the implementation process involves a wide array of stakeholders, each with potentially conflicting interests and differing visions of success, complicating consensus-building and policy execution [[35], [36], [37], [38]]. Moreover, the dynamic environments in which policies operate—shaped by shifting economic conditions, political climates, and technological advancements—necessitate adaptable and scalable policy solutions that respond to evolving circumstances. The challenge is further compounded by difficulties in measuring and evaluating the impacts of policies, especially those aimed at achieving long-term and broad societal goals. Therefore, effective ecological policy implementation in Ayeyarwady demands innovative, integrative approaches that accommodate ongoing changes and engage diverse stakeholders in continual feedback loops to refine and adjust policy directives as necessary. Recognizing and addressing these complexities is crucial for crafting effective and resilient policies.

In summary, the challenges identified in the Ayeyarwady River study reflect a microcosm of global policy implementation issues, particularly in developing economies. The study's insights into the specific barriers at the local level provide valuable additions to the existing literature by highlighting the importance of context-specific strategies in policy implementation, thus enriching the discourse on sustainable development and environmental governance.

### 7.1 Recommendations based on integrated knowledge systems

To effectively contribute to marine safety, the study recommends that:

1. To enhance policy implementation effectiveness, Hudson et al. [20] suggest strengthening knowledge brokerage to bridge information gaps between policymakers and practitioners. The Ayeyarwady study supports this, illustrating the need for continuous and constructive feedback mechanisms that incorporate local insights into national policymaking, thus ensuring that policies are well-informed and tailored to meet local needs effectively.
2. Since many farmers do not understand sustainability and symbiosis between agriculture and ecological safety, all relevant ministries should collaborate to reach out to the farmers deliberately. Repetitive awareness messages will eventually lead to significant mindset change.
3. All stakeholders must continue to hold open discussions about the best possibilities for sustainable development in the Ayeyarwady Basin.
4. To enhance the effectiveness of environmental policies, it is crucial to develop strategies that holistically address the cultural, economic, and political factors influencing policy implementation. This involves:
5. Designing policy incentives that are culturally resonant and economically beneficial can help align farmer behaviors with environmental goals. For example, subsidies for sustainable practices, premiums for eco-friendly products, or economic benefits linked to compliance can motivate farmers to change their practices.
6. Ensuring political stability and governance practices that are informed by and adapted to local cultural contexts can enhance trust and cooperation in the community. This may involve localized policymaking processes that include farmer input and are sensitive to local socio-economic conditions.
7. Integrating approaches that consider cultural norms, economic conditions, and political realities can lead to more robust and resilient policy frameworks. These should involve cross-sector collaboration that leverages local knowledge and aligns policy goals with local needs and values.

Implementing an environmental preservation policy is described as a deliberate and complex process that seeks to influence both the agricultural and education sectors significantly. The implementation is multifaceted, involving stakeholders at various levels who can impact the system according to specific policy goals. This process is also highly contextual, influenced by multiple societal factors, including culture, demographics, politics, and economic conditions. These elements shape how agricultural and educational systems respond to and integrate policy changes. The policy aims to be transformative, intending to modify existing practices to enhance ecological sustainability and better adapt agricultural and educational frameworks to meet evolving societal needs and pressures.

## 8 Limitations to this study and future students

Despite the valuable insights garnered from this research, which involved a sample of 45 participants in Myanmar, certain limitations must be acknowledged. The sample size, while providing a substantial basis for analysis, may constrain the generalizability of the findings. To enhance the robustness and breadth of understanding regarding the relationship between governmental environmental regulations and sustainable economic development within the agricultural sector, future research should consider incorporating a more extensive sample size. This expansion should include a broader demographic representation across various regions of Myanmar, utilizing diverse methodological approaches to capture a wider array of perspectives and experiences.

Furthermore, comparative studies in similar agricultural and environmental contexts in other countries would significantly augment the generalizability and applicability of the findings. Such cross-contextual analyses could elucidate best practices and offer valuable comparative insights that transcend local specificities, thereby contributing to a more comprehensive understanding of effective policy implementation.

Additionally, implementing longitudinal studies to monitor changes in stakeholder awareness and understanding over time would provide profound insights into the enduring impact and efficacy of policy interventions. These longitudinal analyses would capture the dynamic evolution of stakeholder perspectives and the sustained influence of policies, thereby informing more adaptive and resilient strategies for future policy design and implementation. By continuously tracking these changes, researchers can better address the complex, evolving challenges in policy implementation and develop more nuanced, effective interventions that are responsive to the shifting landscape of stakeholder needs and environmental conditions.

## 9 Limitations to this study and future students

This study did not receive any funding.

## Data availability statement

The authors do not have the permission to share data.

## Approval of the submission

All authors approved this submission.

## CRediT authorship contribution statement

**Lazarus Obed Livingstone Banda:** Writing – review & editing, Writing – original draft, Visualization, Validation, Supervision, Software, Resources, Project administration, Methodology, Investigation, Formal analysis, Data curation. **Chigonjetso Victoria Banda:** Visualization, Validation, Resources, Methodology, Data curation. **Jane Thokozani Banda:** Writing – review & editing, Visualization, Supervision, Resources, Project administration, Data curation, Conceptualization. **Thin Thin Hlaing:** Resources, Investigation, Conceptualization. **Eretia Mwaene:** Writing – review & editing, Visualization, Investigation, Data curation, Conceptualization.

## Declaration of competing interest

The authors declare that they have no known competing financial interests or personal relationships that could have appeared to influence the work reported in this paper.
